# Efficacy and Safety of RelabotulinumtoxinA, a New Ready-to-Use Liquid Formulation Botulinum Toxin: Results From the READY-1 Double-Blind, Randomized, Placebo-Controlled Phase 3 Trial in Glabellar Lines

**DOI:** 10.1093/asj/sjae131

**Published:** 2024-06-24

**Authors:** Sachin M Shridharani, Amir Moradi, Lisa Donofrio, Michael H Gold, Brian Biesman, Melissa Chiang, Rosalyn George, Kristel Polder, Nowell Solish, Schwarcz, Xiaoming Lin, Eva Axén, Inna Prygova

## Abstract

**Background:**

RelabotulinumtoxinA (RelaBoNT-A, Galderma, Uppsala, Sweden) is an innovative, ready-to-use liquid botulinum toxin A, produced with PEARL (precipitation-free extraction and activity-preserving refined liquid) manufacturing technology, which yields a potent, complex-free formulation.

**Objectives:**

In the READY-1 study, efficacy and safety outcomes following a single RelaBoNT-A treatment for glabellar line correction were examined.

**Methods:**

Adults with moderate to severe glabellar lines received RelaBoNT-A (50 U) or placebo in a 3:1 randomized, 6-month, phase 3, multicenter, double-blind study. Primary endpoints (examined at Month 1, maximum frown) comprised the composite ≥2-grade response, defined as ≥2-grades improvement from baseline on concurrent investigator (glabellar line investigator live assessment; GL-ILA) and participant (glabellar line subject live assessment; GL-SLA) severity scales (US endpoint), and the investigator-reported responder rate for participants scored as 0 (none) or 1 (mild) (GL-ILA scale only; EU endpoint). Participant satisfaction and treatment-emergent adverse events (TEAEs) were reported.

**Results:**

Overall, 297 adults were randomized and treated. Month 1 composite ≥2-grade responder rate was 82.9% (RelaBoNT-A, *n* = 199) vs 0% (placebo, *n* = 67; *P* < .001). Month 1 investigator-reported none or mild responder rate was 96.3% (RelaBoNT-A) vs 4.5% (placebo; *P* < .001). GL-ILA scores remained higher with RelaBoNT-A (23.6% [none or mild]; 58.1% [≥1-grade improvement]) vs placebo (1.5%; 10.4%, respectively) through Month 6 (*P* < .001). In the Kaplan-Meier analysis, 75% still showed GL-ILA and GL-SLA improvements from baseline at 169 days (end of study). Participants reported onset of effect from Day 1 (39%) and satisfaction with natural-looking results (96.8%; Month 1). RelaBoNT-A–related TEAEs were low (3.6%) and typically mild.

**Conclusions:**

A single RelaBoNT-A treatment was effective and demonstrated a favorable safety profile. RelaBoNT-A provided significant improvements in glabellar line severity, high satisfaction, rapid onset, and enduring effectiveness throughout the 6-month study period.

**Level of Evidence: 1:**

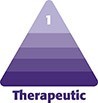

Botulinum toxin type A (BoNT-A) treatments are widely administered for the aesthetic correction of facial lines associated with aging, including glabellar lines, lateral canthal lines, and frontal horizontal forehead lines.^1-8^ Following injection, most BoNT-A treatments provide significant aesthetic improvements for 3 to 5 months.^2-7^ Available products on the market are generally produced with precipitation for initial purification steps and are lyophilized with excipients such as human serum albumin to stabilize the final product.^9-13^ The resulting formulations require reconstitution with saline, which can introduce reconstitution-related errors in dosing and contamination of the final injected product.^9-13^ Ready-to-use BoNT-A formulations have been shown to provide multiple benefits for healthcare practitioners: beyond the obvious convenience and time-saving factor, ready-to-use formulations also bring peace of mind to clinic staff by eliminating any potential reconstitution-related errors.^14^

RelabotulinumtoxinA (RelaBoNT-A, Galderma, Uppsala, Sweden) is an innovative, complex-free, ready-to-use BoNT-A that is currently in development.^15^ It is provided at a consistent concentration of 100 U/mL, which enables simple dosing and unit conversion from the volume injected to the dose administered. Units for RelaBoNT-A cannot be directly compared to those of other BoNT-A products, because the products go through different, proprietary potency testing assays to confirm efficacy and safety of the final product. RelaBoNT-A is manufactured with a proprietary process that yields a pure toxin formulation that is animal and human origin free, contains no accessory proteins, and is produced in an ISO 14001 and carbon-neutral certified factory, thereby offering an alternative option for potential clients.^15,16^ The absence of complexing proteins has been highlighted as potentially decreasing the long-term immunogenic risk due to the decreased protein load.^17^

RelaBoNT-A is produced with precipitation-free extraction and activity-preserving, refined liquid (PEARL) manufacturing technology. The PEARL technology starts with cultivation of a proprietary *Clostridium botulinum* type A1 strain, from which BoNT-A is purified with multiple diafiltration and chromatography steps that include ion-exchange and size-exclusion chromatography. Each stage in the process is designed to keep the core botulinum toxin protein in liquid suspension to maintain its original conformation, minimizing any denaturation or unfolding that could occur during state changes and preserving the activity of the core botulinum protein.^16^

Here, we report results from the phase 3, double-blind, placebo-controlled Relabotulinumtoxin Aesthetic Development Study-1 (READY-1) trial, in which we aimed to examine the efficacy and safety of a single 50-unit (U) RelaBoNT-A treatment when administered for the improvement of moderate to severe glabellar lines in adults.

## METHODS

### Study Design

A 6-month, phase 3, multicenter, randomized, double-blind, placebo-controlled study was conducted at 10 investigational centers across the United States and Canada between February 2020 and January 2021 (clinicaltrials.gov #NCT04249583). The study was conducted in accordance with the principles of the Declaration of Helsinki (1964) and subsequent amendments and the International Council for Harmonization of Technical Requirements for Pharmaceuticals for Human Use good clinical practice guidelines. Participants provided written informed consent, and ethical approval was obtained from each relevant institutional review board. Participants attended study visits at screening, baseline, Day 7, Day 14, Month 1, and then monthly through Month 6. Due to the ongoing COVID-19 pandemic, study investigators were given permission to conduct remote study visits by video or telephone.

### Study Population

The READY-1 study included male and female adults (aged 18 years or older) with moderate to severe glabellar lines. Glabellar line severity was assessed at maximum frown by both the glabellar line investigator live assessment (GL-ILA) photographic scale and the glabellar line subject live assessment (GL-SLA) static categorical scale, both of which had a 4-point grading system: 0 (none), 1 (mild), 2 (moderate), and 3 (severe).

Participants were excluded from the study if they had known allergy or sensitivity to any component of RelaBoNT-A (or any botulinum toxin serotype), had received facial treatment with botulinum toxin (within 9 months) or anticipated a need for botulinum toxin treatment (of any serotype) during the study period. Other exclusion criteria encompassed previous use of hyaluronic acid (within 6 months) or any aesthetic treatments in the glabellar area within the previous 12 months. Individuals were excluded if they had marked facial asymmetry, excessive dermatochalasis or periocular or eyebrow asymmetry, an inability to substantially lessen glabellar lines by physical spreading apart, or excessive skin laxity in the treatment or periorbital area.

### Study Treatment

RelaBoNT-A (100 U/mL) and placebo (buffered solution) were supplied as sterile solutions for injection, stored at 2° to 8°C (36°-46°F). At baseline (Day 0), participants were randomized 3:1 (stratified by study center) to receive either RelaBoNT-A (total dose: 50 U) or placebo, given as 0.1-mL/10-U injections at 5 prespecified sites in the glabellar region (1 point in the procerus muscle and 2 points in each corrugator supercilia muscle). All treating investigators were trained in the administration technique before the study start. Treatment was assigned within the electronic data capture system, according to a randomization list that was computer-generated before the study start by an unblinded statistician. Double-blinding was maintained with utilization of identical vials for placebo and RelaBoNT-A and by restricting access to the randomization list until after database lock.

### Efficacy and Safety Endpoints

The primary endpoint described in the study protocol was the composite ≥2-grade responder rate at Month 1. This endpoint was designed to fulfill US FDA requirements. A composite responder was defined as a participant who achieved a glabellar line severity improvement of at least 2 grades from baseline at maximum frown on both the GL-ILA and GL-SLA scales concurrently. A separate statistical analysis plan (SAP) was created for the EU (and other regions), in which the primary endpoint was investigator-reported responder rate at Month 1. According to EU requirements, responders were defined as those participants who achieved a glabellar line severity score of 0 (none) or 1 (mild) at maximum frown on the GL-ILA scale only. This parameter was also listed as a key secondary endpoint within the US SAP/study protocol.

Other endpoints (secondary and/or exploratory) assessed (at maximum frown) at all posttreatment visits included the responder rate among participants achieving a score of 0 (none) or 1 (mild) with the GL-ILA and GL-SLA scales separately, responder rates for participants with ≥1-grade improvement, the time to onset of treatment effect (based upon data from participant diary cards), the time to loss of a score of 0 (none) or 1 (mild) on both the GL-ILA and GL-SLA scales concomitantly, and the time to return to baseline score or worse (among those achieving severity scores of 0 or 1 during the study period) on both the GL-ILA and GL-SLA scales concomitantly. Participant satisfaction was appraised with the validated Facial Lines Treatment Satisfaction Questionnaire (FLTSQ).

Safety endpoints included the incidence and severity of reported treatment-emergent adverse events (TEAEs). Blood samples were taken at baseline (before treatment) and Month 6 (or in cases of early termination from the study) and tested for blood chemistry and hematology abnormalities. Other examinations assessed vital signs and electrocardiogram (ECG).

### Statistical Analysis

All analyses were conducted with the SAS system (Version 9.4). Confidence intervals (CIs) and *P* values were 2-sided and performed at a significance level of 5%, unless otherwise specified. Based on the primary endpoint of composite responder rate at Month 1 and historical abobotulinumtoxinA data, sample size calculations showed that inclusion of 300 participants (225 in the RelaBoNT-A group and 75 in the placebo group) would have 99% power to detect difference between the treatment groups with a 2-sided test at a 5% significance level (assuming a 5% dropout rate). Analysis for the primary efficacy endpoints was based on the modified intention-to-treat (mITT) population, comprising all participants who were randomized and dispensed the study product with the exception of those who had a photographic and categorical scale Month 1 assessment by remote visit. Multiple imputation was employed for missing values relating to the primary efficacy outcomes.

Analysis for all other efficacy variables was based on the intention-to-treat (ITT) population, comprising all participants who were randomized and dispensed the study product, with remote visits excluded for GL-ILA analyses. The Cochran-Mantel-Haenszel (CMH) test (stratified by region) was employed to compare outcomes between the RelaBoNT-A and placebo groups for each of the endpoints concerning responder rate for participants achieving a score of 0 (none) or 1 (mild), ≥1-grade improvement from baseline (with the GL-ILA and GL-SLA scales separately), and ≥2-grade improvement from baseline (with the GL-ILA and GL-SLA scales separately). Kaplan-Meier analysis estimated the median time to return to baseline score or to loss of 0 (none) or 1 (mild) score on both GL-ILA and GL-SLA scales concurrently. Safety assessments were conducted with the safety population, comprising all participants who were administered the RelaBoNT-A or placebo.

## RESULTS

### Study Population

In total, 300 participants were randomized and 297 went on to receive RelaBoNT-A (*n* = 223) or placebo (*n* = 74) and were included in the ITT population. A vast majority of the randomized participants, 289 participants (96%), completed the Month 1 visit and 276 (92%) completed the study including Month 6. Due to the COVID-19 pandemic, remote assessments were performed for some participants. Those assessed remotely at Month 1 (*n* = 31) were excluded from the mITT population, resulting in 266 participants included in the primary endpoint analysis: RelaBoNT-A (*n* = 199) and placebo (*n* = 67). Baseline participant demographics and characteristics are shown in Table 1. Most participants were female (90%) and White (84%). Overall, 8% were Black/African American, 4% were Asian, and 5% were reported in other race categories. All Fitzpatrick skin types were represented, the most common types being II (34%) or III (40%). Mean (standard deviation [SD]) age was 47.6 (12.1; range 21–81) years, and the majority of participants were reported to have severe glabellar lines when assessed with the GL-ILA 4-point photographic scale (78.5% [RelaBoNT-A]; 82.4% [placebo]) and GL-SLA static 4-point categorical scale (68.6% [RelaBoNT-A]; 63.5% [placebo]). Slightly less than half of the participant population (45%) was toxin-naïve.

**Table 1.** Baseline Demographics and Characteristics (ITT Population)

Category	RelaBoNT-A (N = 223)	Placebo (N = 74)	Total (N = 297)
Age at baseline (years)			
Mean (standard deviation)	47.6 (12.41)	47.6 (11.19)	47.6 (12.10)
Median	49.0	48.0	48.0
Minimum, maximum	21, 81	27, 72	21, 81
Sex, *n* (%)			
Female	203 (91.0)	65 (87.8)	268 (90.2)
Male	20 (9.0)	9 (12.2)	29 (9.8)
Race, *n* (%)			
White	186 (83.4)	62 (83.8)	248 (83.5)
Black/African American	17 (7.6)	7 (9.5)	24 (8.1)
Asian	9 (4.0)	2 (2.7)	11 (3.7)
American Indian or Alaska Native	4 (1.8)	0	4 (1.3)
Native Hawaiian or Other Pacific Islander	0	0	0
Other	8 (3.6)	4 (5.4)	12 (4.0)
Ethnicity, *n* (%)			
Hispanic or Latino	21 (9.4)	10 (13.5)	31 (10.4)
Not Hispanic or Latino	202 (90.6)	64 (86.5)	266 (89.6)
Fitzpatrick skin type, *n* (%)			
I	2 (0.9)	0	2 (0.7)
II	84 (37.7)	17 (23.0)	101 (34.0)
III	84 (37.7)	34 (45.9)	118 (39.7)
IV	28 (12.6)	16 (21.6)	44 (14.8)
V	11 (4.9)	1 (1.4)	12 (4.0)
VI	14 (6.3)	6 (8.1)	20 (6.7)
Previous botulinum toxin use, *n* (%)			
Naïve	98 (43.9)	36 (48.6)	134 (45.1)
Non-naïve	125 (56.1)	38 (51.4)	163 (54.9)
GL-ILA 4-point photographic scale at maximum frown, *n*/N (%)			—
None	0/223	0/74	
Mild	0/223	0/74	
Moderate	48/223 (21.5)	13/74 (17.6)	
Severe	175/223 (78.5)	61/74 (82.4)	
GL-SLA static 4-point categorical scale at maximum frown, *n*/N (%)			—
None	0/223	0/74	
Mild	0/223	0/74	
Moderate	70/223 (31.4)	27/74 (36.5)	
Severe	153/223 (68.6)	47/74 (63.5)	

N = number of participants in intention-to-treat population; *n* = number of participants in specific category. GL-ILA, glabellar lines investigator live assessment; GL-SLA, glabellar lines subject live assessment; ITT, intention to treat; RelaBoNT-A, RelabotulinumtoxinA.

### Efficacy Outcomes

**Figure 1.**
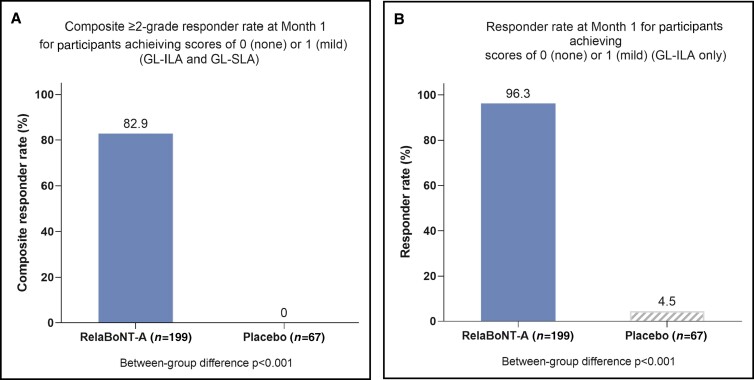
Primary efficacy outcomes (mITT population, with multiple imputation to handle missing values). (A) Composite ≥2-grade responder rate at Month 1, in which responder was defined as participants achieving at least a 2-grade improvement from baseline at maximum frown based on concomitant investigator (GL-ILA) and participant (GL-SLA) scales (US SAP). (B) Investigator-reported responder rate at Month 1, in which responder was defined as participants achieving a glabellar line severity score of 0 (none) or 1 (mild) from baseline at maximum frown with the GL-ILA scale (EU SAP). GL-ILA, glabellar line investigator live assessment; GL-SLA, glabellar line subject live assessment; mITT: modified intention to treat (population comprising all participants who were randomized and dispensed the study product with the exception of those who had a photographic and categorical scale Month 1 assessment by remote visit); RelaBoNT-A, RelabotulinumtoxinA; SAP, statistical analysis plan.

**Figure 2.**
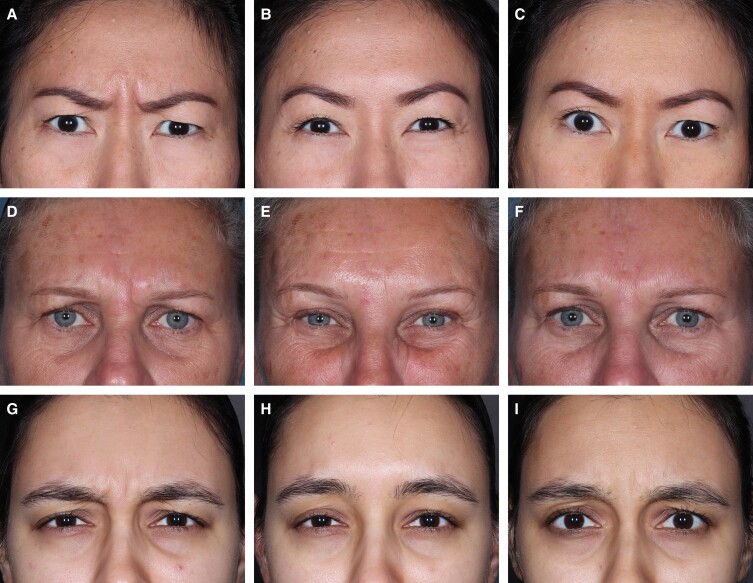
Photographic outcomes for 3 participants treated with RelaBoNT-A who achieved a glabellar line severity score of 0 (none) or 1 (mild) and a ≥ 2-grade improvement from baseline on the GL-ILA scale (maximum frown). Images A-C: a 42-year-old Asian female participant at (A) baseline, GL-ILA = 3; (B) Month 1, GL-ILA = 0; and (C) Month 6, GL-ILA = 1. Images D-F: a 56-year-old White female participant at (D) baseline, GL-ILA = 3; (E) Month 3, GL-ILA = 1; and (F) Month 6, GL-ILA = 1. Images G-I: a 24-year-old American Indian or Alaska Native female participant at (G) baseline, GL-ILA = 3; (H) Day 7, GL-ILA = 0; and (I) Month 6, GL-ILA = 1. GL-ILA, glabellar line investigator live assessment; RelaBoNT-A, RelabotulinumtoxinA.

The primary endpoints were met according to both the US and EU definitions (Figure 1A, B). The composite ≥2-grade responder rate at Month 1 on both GL-ILA and GL-SLA scales concurrently (US primary endpoint) was significantly higher for those in the RelaBoNT-A (82.9%) group, vs placebo (0%) (*P* < .001; mITT population). Month 1 investigator-reported responder rate, defined as participants who achieved a glabellar line severity score of 0 (none) or 1 (mild) at maximum frown on the GL-ILA scale only (EU primary endpoint), was 96.3% in the RelaBoNT-A group and 4.5% in the placebo group (*P* < .001; mITT population, multiple imputation of missing values). Figure 2 shows photographic outcomes for 3 participants achieving glabellar line severity scores of 0 (none) or 1 (mild) and ≥2-grade improvement from baseline at maximum frown on the GL-ILA scale.

**Figure 3.**
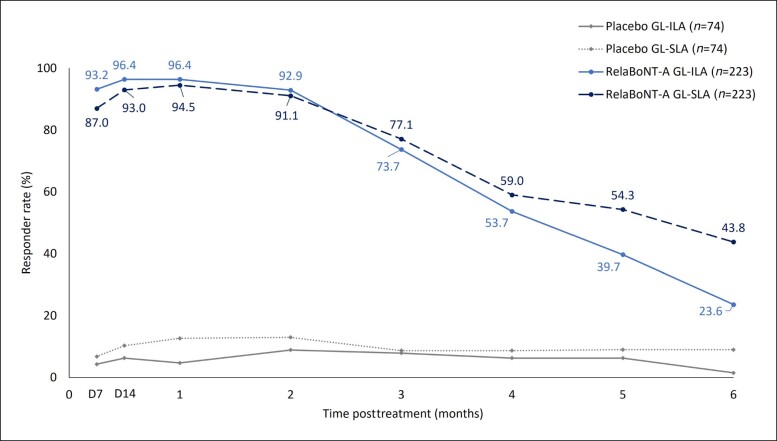
Investigator-reported and participant-reported glabellar line responder rate during the 6-month study period (maximum frown; ITT population, using observed cases only with no imputation for missing values). Responders were defined as participants achieving a glabellar line severity score of 0 (none) or 1 (mild) at maximum frown based on the GL-ILA 4-point photographic scale and the GL-SLA 4-point categorical scale, respectively. Responder rates were significantly greater at all study visits with RelaBoNT-A treatment compared with placebo when assessed with either the GL-ILA or GL-SLA scales (*P* < .001). Remote assessments were excluded for GL-ILA. D, Day; GL-ILA, glabellar line investigator live assessment; GL-SLA, glabellar line subject live assessment; ITT, intention to treat; RelaBoNT-A, RelabotulinumtoxinA.

In total, 96.4% and 23.6% of participants treated with RelaBoNT-A were rated as 0 (none) or 1 (mild) on the GL-ILA scale at Months 1 and 6, respectively (ITT population, observed cases, Figure 3). Participant-reported responder rates (GL-SLA scale) were 94.5% and 43.8% in the RelaBoNT-A group at Months 1 and 6, respectively. Scores of 0 (none) or 1 (mild) were maintained for more than half of RelaBoNT-A–treated participants through Month 4 according to the GL-ILA scale and through Month 5 by the GL-SLA scale. Responder rates were significantly higher in the RelaBoNT-A group compared with the placebo group at all study visits when rated with either the GL-ILA or GL-SLA scale (*P* < .001; ITT population). Placebo group responder rates were <9% on the GL-ILA scale and ≤13% on the GL-SLA scale throughout the 6-month study period.

**Figure 4.**
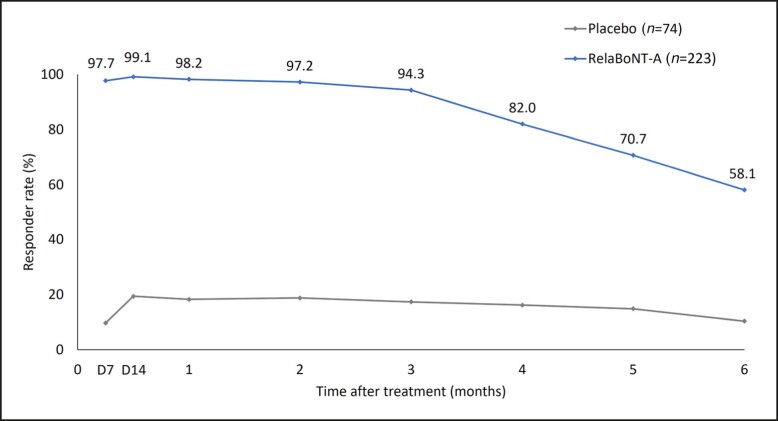
Glabellar line investigator live assessment responder rate for participants achieving ≥1-grade improvement from baseline (maximum frown; observed cases, ITT population). Responder rates were significantly greater at all study visits with RelaBoNT-A treatment compared with placebo (*P* < .001). D, Day; ITT, intention to treat; RelaBoNT-A, RelabotulinumtoxinA.

Responder rates were high (98.2%) at Month 1 for participants treated with RelaBoNT-A who achieved ≥1-grade improvement on the GL-ILA scale from baseline at maximum frown. The responder rate for ≥1-grade improvement was maintained above 90% from Day 7 through Month 3 and was 58.1% at Month 6 (Figure 4). GL-ILA ≥1-grade improvement was significantly higher at all study visits with RelaBoNT-A treatment compared to the placebo group (*P* < .001; ITT population), in which responder rates ranged between 9.7% and 19.4% during the study period.

**Figure 5.**
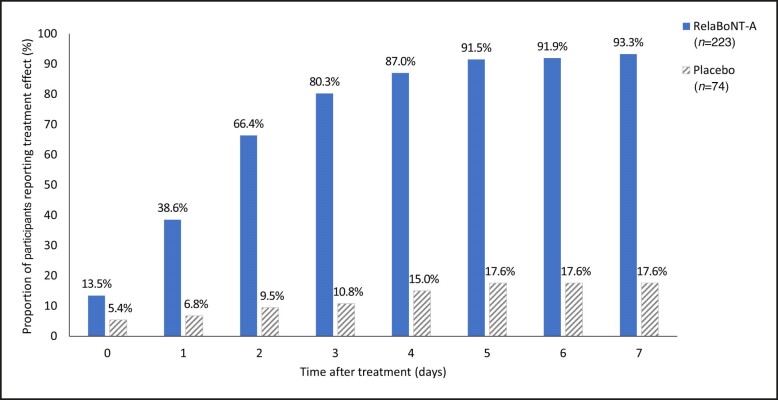
Participant-reported onset of treatment effect in diary cards following administration of RelaBoNT-A or placebo (observed cases; ITT population). Treatment was administered on Day 0. ITT, intention to treat; RelaBoNT-A, RelabotulinumtoxinA.

**Figure 6.**
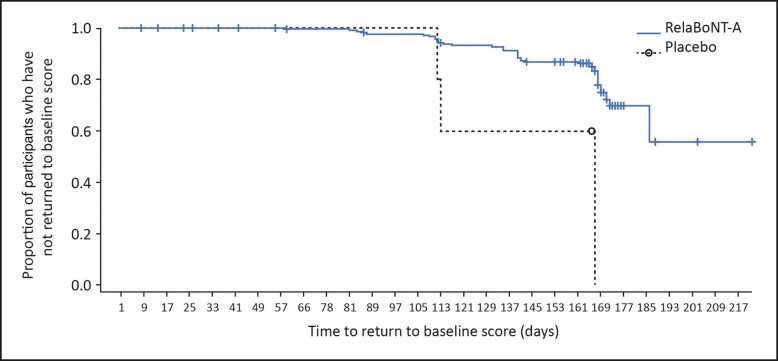
Kaplan-Meier plot of time to return to baseline score based on concurrent investigator- and participant-reported assessments (ITT population). Participants who had not returned to baseline at the end of the study were censored at their last date in the study, meaning that the analysis accounted for the fact that these participants' return to baseline was at some unknown time after their last study visit. Note that some participants had their Month 6 visit well after Day 169 (up to Day 217) resulting in event/censor time after Day 169. ITT, intention to treat; RelaBoNT-A, RelabotulinumtoxinA.

Participant diary card data showed that 39% reported treatment efficacy to be visible by Day 1, and the median time to onset of treatment effect was 2 days (Figure 5). Median time to loss of 0 (none) or 1 (mild) score based on the concurrent use of the GL-ILA and GL-SLA scales was 168 days (24 weeks/6 months) after RelaBoNT-A-treatment (ITT population). The median time to return to baseline GL-ILA and GL-SLA score was >6 months (beyond the end of the study period). Based on Kaplan-Meier estimates, 75% of participants in the RelaBoNT-A group had not returned to baseline on concurrent scales at 169 days (>24 weeks; Figure 6).

FLTSQ assessments revealed that participant satisfaction with treatment outcome was high at Month 1 (96.8%) through Month 6 (85.6%) in the RelaBoNT-A group. Around the time of peak efficacy (Month 1), 96.8% of participants reported being satisfied with how natural their face looked after RelaBoNT-A treatment, and satisfaction was maintained by the majority (88.1%) through Month 6. At Month 1 (98.1%) and at Month 6 (89.5%), most participants indicated that they would like to receive the same treatment again. At Month 1, 97.7% said that they would recommend the treatment to others and 90.0% stated that this was still the case at Month 6.

### Safety Endpoints

TEAEs were reported in 19.4% (43 participants) of RelaBoNT-A recipients and 18.7% (14 participants) of the placebo group. Overall, 3.6% (8 participants) of RelaBoNT-A recipients each had 1 TEAE that was considered related to the study treatment or injection procedure. Treatment-related TEAEs are summarized in Table 2. Most were mild in intensity and none was severe. In the RelaBoNT-A group, 2.3% (5 participants) reported headache, (2 mild, 3 moderate); 0.9% (2 participants) had mild eyelid ptosis (1 event each); and 0.5% (1 participant) experienced a single event of mild injection site bruising. All treatment-related TEAEs occurred within the first month and resolved within 2 months. No TEAEs were reported to be related to placebo. During the study period, RelaBoNT-A treatment was not associated with any clinically meaningful mean changes from baseline related to hematology, blood chemistry, vital sign, or ECG parameters. There were no reports of remote toxin spread or hypersensitivity to RelaBoNT-A.

**Table 2.** Summary of Reported Treatment-related Adverse Events (Safety Population)

System organ class Preferred term	RelaBoNT-A (*n* = 222)		Placebo (*n* = 75)	
	*n* (%)	Events	*n* (%)	Events
Participants with at least 1 related TEAE	8 (3.6)	8	0	0
Nervous system disorders	5 (2.3)	5	0	0
Headache	5 (2.3)	5	0	0
Eye disorders	2 (0.9)	2	0	0
Eyelid ptosis	2 (0.9)	2	0	0
General disorders and administration site conditions	1 (0.5)	1	0	0
Injection site bruising	1 (0.5)	1	0	0

N = number of subjects in safety population, *n* = number of subjects in specific category. RelaBoNT-A, RelabotulinumtoxinA; TEAE, treatment-emergent adverse event.

## DISCUSSION

A single RelaBoNT-A (50 U) treatment provided statistically significant improvements in line severity through 6 months, compared with placebo, among individuals with moderate to severe glabellar lines. Investigator assessments revealed that the majority of participants (∼80%) treated with RelaBoNT-A had severe glabellar lines at baseline, and yet responder rates were high from Month 1 across each of the efficacy endpoints evaluated and were sustained through Month 6, with the majority of participants maintaining improvements at the end of the study period (24 weeks) (Figures 1, 3, 4, 6). Treatment was also fast-acting, with onset reported from Day 1 by 39% of participants (Figure 5). RelaBoNT-A was well tolerated and satisfaction was rated highly throughout the study. These outcomes suggest that, in addition to the convenience and potential time-saving effect of a ready-to-use formulation for the injector,^14^ RelaBoNT-A potentially offers clinical advantages for the patient in glabellar lines compared to other available BoNT-A treatments.^9-13^ With a rigorous composite responder approach, the majority (83%) of RelaBoNT-A-treated participants achieved at least 2-grade improvements from baseline as well as scores of 0 (none) or 1 (mild) at Month 1 (maximum frown) when assessed with investigator- and participant-reported scales concurrently (Figure 1A). Previously approved toxins in the US (abobotulinumtoxinA, incobotulinumtoxinA, prabotulinumtoxinA, and daxibotulinumtoxinA) have reported composite responder rates of 48% to 74%.^9-11,13^ Almost all participants (98.2%) demonstrated improvements of ≥1 grade from baseline at Month 1 (Figure 4) with RelaBoNT-A treatment, and 96.4% achieved scores of 0 (none) or 1 (mild), as judged by investigators (Figure 3). Nonresponders to BoNT-As exist due to preexisting neutralizing antibodies;^17^ however, in the present study very few participants failed to respond to treatment.

Diary card data showed that onset of treatment effect was rapid with RelaBoNT-A. Almost 40% of RelaBoNT-A recipients reported efficacy to be evident just 1 day after treatment (Figure 5). Median time to onset of treatment effect was 2 days, which is aligned with or faster than efficacy onset data reported for other BoNT-A products that are already licensed for the aesthetic improvement of glabellar lines (typically 1-4 days).^2-7,18-21^ Onset of treatment effect has been reported at days 2 to 7 for onabotulinumtoxinA, daxibotulinumtoxinA, and prabotulinumtoxinA.^22-29^

Treatment was long-lasting, with the majority of participants (58.1%) still showing ≥1-grade improvements from baseline at Month 6 in the investigator assessments (GL-ILA, Figure 4) and most participants (approximately 75%) not having returned to baseline severity according to both GL-ILA and GL-SLA scales at the end of the study period, based on the Kaplan-Meier estimates (Figure 6). The long duration of effect is potentially associated with the high potency of the innovative and pure RelaBoNT-A formulation, which contains no accessory proteins due to the manufacturing and purification processes.^15,16^ While most available *C. botulinum* neurotoxins are manufactured with precipitation purification techniques that require lyophilization to stabilize the final product (and therefore reconstitution in saline before administration), the PEARL technology applied to make RelaBoNT-A is precipitation-free and designed to maintain the core toxin protein in liquid suspension in its original conformation.^9-16^ The activity of the botulinum protein is subsequently retained, and there is no requirement for lyophilization or reconstitution before use.^15,16^ Preservation of the high purity core toxin protein in the RelaBoNT-A liquid formulation may assist in delivering the rapid onset of treatment effect and high responder rates that were sustained throughout our study.

Participant satisfaction was high throughout the study period (≥85.6%) and reflected the ≥1-grade response rate from Month 1 through Month 6. Most participants (>96%) considered treatment results to look natural around the time of peak effect (Month 1). Throughout the 6-month study period, participants (>89%) indicated that they would like to receive the same treatment again and that they would recommend RelaBoNT-A treatment to others (>90%).

Safety results indicated that RelaBoNT-A treatment was well tolerated in the study population, with predominantly mild treatment-related TEAEs reported. Eyelid ptosis (mild) was seen in less than 1% of RelaBoNT-A recipients, and there were no reports of pain related to study treatment (Table 2). These results provide strong evidence in support of RelaBoNT-A treatment tolerability when utilized for correction of moderate to severe glabellar lines in adults and are in line with safety profiles of other BoNT-As previously approved for glabellar lines in the US.^9-13^ Moreover, the volume of RelaBoNT-A product for each injection point (0.1 mL) for glabellar line treatment is the same as the injection volume for several powder BoNT-As approved for glabellar line treatment,^10-12^ suggesting that a precise clinical effect can be obtained in the intended muscle with the planned dose and concentration.

One limitation of this study was that the final visit was at Month 6, and because the majority of participants treated with RelaBoNT-A had not returned to baseline severity at the end of the study period (Figure 6) future studies would benefit from a longer follow-up period to fully assess the longevity of a single RelaBoNT-A treatment. Another possible weakness was that the study took place during the COVID-19 pandemic, resulting in some assessments being performed remotely. To ensure that these circumstances did not impact the main conclusions, these data were excluded from the primary analysis, and sensitivity analyses then were conducted on the full population to confirm that statistical results were robust (data not shown). In addition, while treatment effectiveness was assessed with a robust approach that encompassed both investigator- and participant-reported data, a 4-point scale may have limited the ability to capture more subtle improvements in glabellar line severity. A wider ranging scale (5-points or more) may have provided a more refined measure of treatment effectiveness. However, the 4-point scales in our study reflect those widely employed and accepted across the literature for the measurement of glabellar line severity following botulinum toxin treatment.^3,18,21-26,28,29^

In summary, the data presented indicate that the high purity, complex-free RelaBoNT-A liquid formulation provides a highly efficacious treatment option for the correction of moderate to severe glabellar lines with a favorable safety profile. A single RelaBoNT-A treatment demonstrated efficacy from Day 1, with results lasting throughout the 6-month study period. The rigorous manufacturing process of producing RelaBoNT-A and preserving the activity of the botulinum toxin protein may explain the clinical efficacy seen in the study, and the ready-to-use formulation offers potential time-saving and peace of mind for physicians who regularly administer BoNT-A formulations in their clinical practice.

## CONCLUSIONS

A single RelaBoNT-A treatment provided significant improvements in glabellar line severity. Efficacy was demonstrated over an extended period, through Month 6 (study end) for the majority of participants, and almost 40% experienced onset of treatment effect within 1 day after injection. RelaBoNT-A was generally well tolerated, and participants reported high satisfaction with the natural appearance of their face. Treatment with RelaBoNT-A may provide a long-lasting, fast-acting ready-to-use treatment for natural-looking aesthetic improvement.
